# Retrosternal Deformations after Coronary Artery Bypass Surgery Using Statistical Shape Analysis

**DOI:** 10.21470/1678-9741-2020-0294

**Published:** 2021

**Authors:** Mehmet Senel Bademci, Gokhan Ocakoglu, Cemal Kocaaslan, Fatih Avni Bayraktar, Kaptaniderya Tayfur, Ebuzer Aydin

**Affiliations:** 1 Department of Cardiovascular Surgery, Istanbul Medeniyet University, Faculty of Medicine, Istanbul, Turkey.; 2 Department of Biostatistics, Bursa Uludag University, Faculty of Medicine, Bursa, Turkey.; 3 Department of Cardiovascular Surgery, Ordu University, Faculty of Medicine, Ordu, Turkey.

**Keywords:** Coronary Artery Bypass, Pulmonary Artery, Sternum, Aorta, Cardiac Surgical Procedures, Emergency Service, Hospital, Surgeons, Tomography

## Abstract

**Introduction:**

In this study, we aimed to evaluate the anatomical deformations of the major vascular structures in the retrosternal area caused by adhesions following coronary artery bypass grafting (CABG).

**Methods:**

This single-center, retrospective study included a total of 40 patients with a previous CABG who were admitted to our emergency unit for any reason and underwent a contrast-enhanced chest computed tomography (patient group) and 40 patients without previous cardiac surgery (control group) between January 2018 and November 2019. The retrosternal area was compared between the groups using the statistical shape analysis method. The distance between the sternum and the ascending aorta and pulmonary artery was measured and anatomical deformations of the retrosternal area were examined.

**Results:**

There was a statistically significant difference in the anatomical structures of the retrosternal area between the patient and control groups (*P*<0.001). The distance from the midsternal line to the highest point of the pulmonary artery was statistically significantly shorter in the patient group, compared to the control group (*P*=0.013). The distance from the sternum to the ascending aorta was also shorter in the patient group, although it did not reach statistical significance (*P*>0.05).

**Conclusions:**

Our study results showed narrowing of the retrosternal area following CABG and a shorter distance from the sternum to the pulmonary artery than the ascending aorta. Based on these findings, surgeons should be cautious about possible injuries in patients requiring cardiac surgery with repeated median sternotomy.

**Table:** 

Abbreviations, acronyms & symbols	
**CABG**	**= Coronary artery bypass grafting**
**COPD**	**= Chronic obstructive pulmonary disease**
**CT**	**= Computed tomography**
**G**	**= Generalizability**
**GT**	**= Generalizability theory**
**SD**	**= Standard deviation**
**TPS**	**= Thin plate spline**

## INTRODUCTION

With the development of operative cardioprotective techniques over the past few decades, cardiac surgery has evolved dramatically and has been widely used to reduce mortality from cardiovascular diseases^[1]^. Median sternotomy is the most frequently used incision in open-heart surgery, and the increased number of operations and life expectancy with an aging population requires reoperations. Following sternotomy, adhesions between the major cardiovascular structures and grafts of the coronary artery bypass grafting (CABG), such as mammary artery or saphenous vein in the retrosternal area, are one of the leading causes of morbidity and mortality for patients requiring resternotomy^[2]^. In the practice of cardiovascular surgery, a variety of incisions can be used through minimally invasive techniques to minimize the risk of morbidity and mortality^[3]^. Preoperative radiological imaging studies are of utmost importance for patients requiring redo cardiac surgery with sternotomy to evaluate adhesions of the retrosternal area and the proximity between the sternum and vital organs and to ensure a safe surgery^[4]^.

In recent years, a considerable number of studies based on the geometric characteristics of organs or organisms investigating the change in the geometric properties of these structures with a specific disease or environmental factors have been reported in the literature^[5,6]^. The statistical shape analysis method is a modern geometric morphometric method that utilizes the shape of organs or organisms as input data. In addition to the interpretation of the difference between shapes, the statistical shape analysis stands out as an auxiliary tool that allows the interpretation of structural differentiation in the organ of interest.

In the present study, we aimed to evaluate the anatomical deformations of the major vascular structures in the retrosternal area caused by adhesions following median sternotomy incision in patients undergoing isolated CABG and to investigate possible advantages of preoperative imaging using statistical shape analysis in patients requiring redo cardiac surgery.

## METHODS

### Study Design and Study Population

This single-center, retrospective study was conducted at Department of Cardiovascular Surgery, Istanbul Medeniyet University, Faculty of Medicine, between January 2018 and November 2019. Contrast-enhanced chest computed tomography (CT) transverse scans of a total of 40 patients with a previous CABG who were admitted to our emergency unit for any reason (patient group) and 40 patients without a previous cardiac surgery (control group) were included in the study. All CT imaging examinations were performed using a multi-slice CT scanner (SOMATOM Plus 4, Siemens Medical Solutions, Forchheim, Germany). The patient group consisted of those with a sternotomy incision, while the control group consisted of those without a sternotomy incision. In each patient, six anatomical landmarks were marked in the transverse slice of thoracic CT at the level of pulmonary artery bifurcation. All CT scans were retrospectively retrieved from the computer-based patient record system. Exclusion criteria were as follows: history of chronic obstructive pulmonary disease (COPD), sternal dehiscence and mediastinitis after cardiac surgery, non-cardiac thoracic surgery, non-coronary cardiac surgery (i.e., valve operations), chest trauma, chest radiotherapy, resternotomy and heart transplantation, ventricular assist device implantation, and correction of congenital heart defects. The distance between the sternum and the vertebral body was calculated to avoid statistical bias. A written informed consent was obtained from each patient. The study protocol was approved by the local Ethics Committee. The study was conducted in accordance with the principles of the Declaration of Helsinki.

### Collection of Two-Dimensional Retrosternal Landmarks

**Fig. 1:**
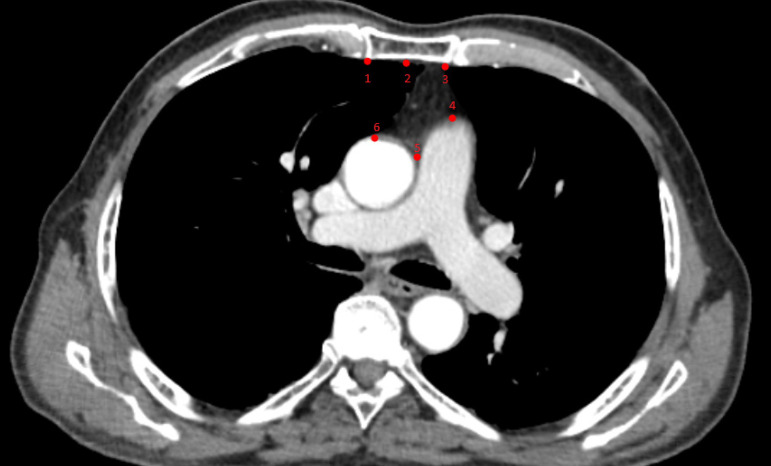
A computed tomography scan showing landmarks markings on retrosternal space at the main pulmonary artery branching level.

Data of the marked regions were collected from two-dimensional digital CT images. Six anatomical landmarks were considered as references obtained in the image corresponding to the same transverse plane (main pulmonary artery branching level) (Figure 1) and marked with the TPSDIG software version 2.04 ((c)2004 by F. James Rohlf). Definitions of the landmarks used in the present study are given in Table 1.

**Table 1:** Definitions of landmarks used in the study.

Landmark number	Description
Landmark 1	Left retrosternal point
Landmark 2	Mid-retrosternal point
Landmark 3	Right restrosternal point
Landmark 4	Highest point of the main pulmonary artery
Landmark 5	Highest junctional point of aorta and main pulmonary artery
Landmark 6	Highest point of the ascending aorta

LM=landmark

### Geometric Morphometric Analysis

#### Statistical Shape Analysis

The shape difference between the patient and control groups was assessed using the Generalized Procrustes Analysis (GPA) method. The Box's M procedure was used to test the equality of variance-covariance matrices. Since the variance-covariance matrices were unequal (*P*=0.002), the James F_J_ test based on a resampling procedure was performed for shape comparisons^[7]^. To obtain the overall measures of shape variability for the groups, we compared the root mean square of Kendall's Riemannian distance (*rho*) with the mean shape. The shape deformation of the retrosternal space from the patient group to the control group was evaluated using the Thin Plate Spline (TPS) analysis, derived from a mathematical model used in computer graphics and applied to morphometrics by Bookstein^[8]^. The points showing the greatest enlargement or reduction labeled as deformation were established through the TPS analysis.

#### Landmark Reliability

The intrarater reliability coefficient was calculated for a two-facet crossed design ('landmark pairs-by rater-by-subject', l x r x s) based on the generalizability theory (GT). Accordingly, the generalizability (G) coefficient refers to the reliability for relative (norm-referenced) interpretations. In this study, all landmarks were marked. Two weeks later, 30 subjects (n=15 patients and n=15 controls) randomly selected from the study population were marked. An analysis was performed to obtain a G reliability coefficient and showed a strong repeatability for patients (G=0.9685) and controls (G=0.9743)^[9]^.

#### Statistical Analysis

Statistical analysis was performed using SPSS for Windows version 23.0 (IBM Corp., Armonk, NY, USA), R version 3.5.1, and PAST version 3.0 statistical software packages. Descriptive data were presented as mean ± standard deviation (SD), median (min-max) or number and frequency. The Shapiro-Wilk test was used to assess the normality of data distribution. The independent samples t-test was performed to compare age and aortic diameter between groups. The Mann-Whitney U test was used to compare sternal, aortic and pulmonary distance between groups. The chi-square test was used to analyze sex distribution between groups. A *P*-value <0.05 was considered statistically significant.

## RESULTS

A total of 40 patients (21 males and 19 females) with previous CABG and 40 control subjects (21 males and 19 females) without previous cardiac surgery were included in this study. The mean age was 61.85±6.56 (range, 47 to 75) years in the patient group and 60.05±15.18 (range, 28 to 86) years in the control group. There was no significant difference in the age and sex of the patients and controls (*P*=0.94 and *P*>0.99, respectively). In addition, no significant difference in the body mass index, sternal-vertebral distance, aortic diameter, and pulmonary artery diameter was observed between groups (Table 2).

**Table 2:** Measurements of patient and control groups.

	Patient group (n=40)	Control group (n=40)	*P*-value
Body mass index, kg/m^2^	25.75±3.17 (19:32)	26.38±3.22 (21:34)	0.384^a^
Aortic diameter, cm	3.60±0.43 (2.50:4.40)	3.49±0.51 (2.50:4.80)	0.292^a^
Sternal-vertebral distance, cm	10.30 (8.90:15.10)	10.50 (7.50:15)	0.675^b^
Pulmonary artery diameter, cm	2.50 (1.80:3.90)	2.50 (1.80:4)	0.546^b^
Mid-retrosternal-aortic distance, cm (LM 2-6)	25.35 (8.10:53.30)	29.30 (17.60:57.50)	0.055^b^
Mid-retrosternal-pulmonary artery distance, cm (LM 2-4)	35.48±10.08 (15.90:58.80)	41.20±10.12 (24:64.40)	0.013^a^

Data are given as mean ± standard deviation or median (min:max), unless otherwise stated.

a Independent samples *t*-test.

b Mann-Whitney U test.

Using the CT slices, the distance from the highest point of the ascending aorta (landmark 6) to the mid-retrosternal point (landmark 2) was shorter in the patient group, although there was no statistically significant difference between the groups (*P*=0.055). However, the distance from the highest point of the pulmonary artery (landmark 4) to the mid-retrosternal point (landmark 2) was statistically significantly shorter in the patient group, compared to the control group (*P*=0.013) (Table 2).

**Fig. 2:**
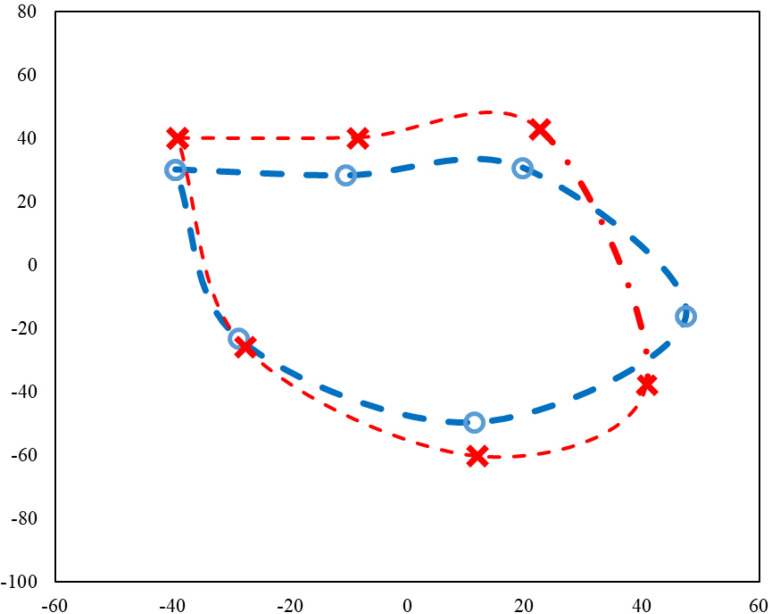
Procrustes mean shapes for images of the retrosternal area of patients (o) and controls (x).

Shape differences and shape changes between the groups were analyzed using the statistical shape analysis method. The Procrustes mean shapes were computed for both groups (Figure 2). Figure 2 shows the preoperative (red lines) and postoperative (blue lines) retrosternal area, indicating narrowing in the postoperative period. Accordingly, there was a statistically significant difference in the shape of the retrosternal area between the groups (*P*<0.001). Based on the evaluation of the retrosternal area according to the shape variation, there were large variations in the patient group, compared to the control group (0.325 *vs*. 0.218, respectively).

**Fig. 3:**
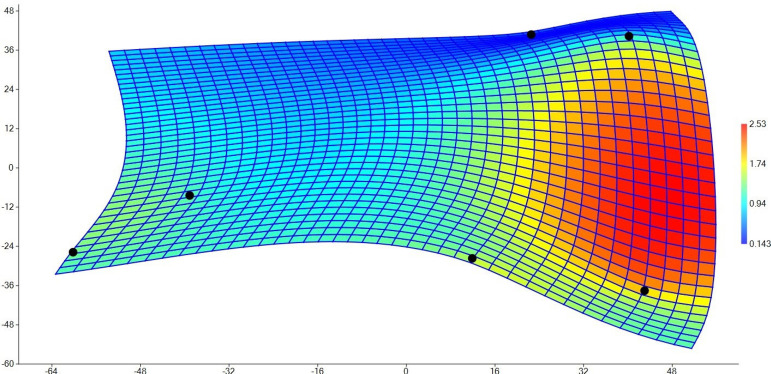
A thin plate spline showing the mean retrosternal space shape deformation for control group versus patient group.

Furthermore, a high rate of deformations in the retrosternal space was observed in the patient group, as assessed by the TPS graph (Figure 3). Figure 3 shows the deformation (distance changes) in the landmarks based on a color scale, indicating that landmarks 3 and 4 statistically significantly approximates each other in the postoperative period. The highest deformation was found between the right retrosternal point (landmark 3) and the highest point of the pulmonary artery (landmark 4). According to the mean shapes, the shape of the retrosternal area, particularly between the right retrosternal point and the highest point of the pulmonary artery, exhibited a significant expansion in the control group, compared to the patient group.

## DISCUSSION

In the present study, we performed a landmark-based statistical shape analysis method to evaluate changes in the shapes of the retrosternal area in CABG patients compared to the controls. The results of our study showed that the shape variability in the retrosternal area was higher in the patient group, compared with the control group. This anatomical deformation can be attributed to the postoperative scar tissue formation, increased pericardial inflammatory response and fibroblast proliferation, and adhesions of the surrounding tissues in this area^[10]^. Similarly, we found that the major anatomical deformation occurred between landmark 3 and landmark 4 in our study. In addition, we measured the distance from the mid-retrosternal point (landmark 2) to the highest point of the main pulmonary artery (landmark 4) and the highest point of the ascending aorta (landmark 6) and observed narrowing in the retrosternal area following CABG and a statistically significantly shorter distance from the sternum to the pulmonary artery in the patient group.

The opening of the pericardial cavity promotes formation of retrosternal adhesions due to the anatomical position, which contracts the right ventricle toward the sternum. This can explain why the main pulmonary artery, which is located at the anatomical continuity of the right ventricle, approaches the sternum significantly, rather than the ascending aorta. In an earlier cadaveric study, Livi et al.^[11]^ assessed the samples of aortic and pulmonary walls for viability and morphological characteristics and reported that the normal configuration of the pulmonary elastica was more sparse, irregular, and fragmented than the normal aortic elastic pattern. It has been shown that the elastic fibers of the vessel wall increase the vascular resistance in the presence of excessive strain, by surrounding the collagen bundles. Similarly, we observed a greater narrowing in the pulmonary artery, rather than the ascending aorta, in the retrosternal area in patients with CABG, which was possibly due to the excessive strain caused by postoperative adhesions.

The right ventricle is the most commonly injured chamber following resternotomy, particularly in patients with pulmonary arterial hypertension^[12]^. In addition, injuries of the major vascular structures following resternotomy can be seen in 1.5% of cases^[13]^. With the introduction of recent technological developments and increased life expectancy with the aging of the population, redo cardiac surgeries have been widely performed, despite high intraoperative mortality rates^[14]^. The primary closure of the pericardium, as much as possible, is therefore of utmost importance for patients requiring redo surgery. A previous study showed that 88% of patients with catastrophic injuries after resternotomy were those in which the pericardium was left open^[15]^. In another study, Boyd et al.^[16]^reported that primary pericardial closure reduced the risk of possible injuries during resternotomy. On the other hand, as the primary closure with the native pericardium or other material such as polytetrafluoroethylene or autologous fascia lata may result in early postoperative compression and eventually cardiac tamponade and/or hemodynamic deterioration, it is still controversial^[17]^.

It is critical not to advance the graft through the anterior surface of the right ventricle during CABG, particularly the right CABG, and to advance the graft through the lateral of the sternal line during left internal mammary artery grafting for the left anterior descending artery in patients requiring resternotomy to minimize graft injuries. Resternotomy during redo surgery may lead to loss of surgical dissection planes and difficult-to-treat injuries due to adhesions and scar tissue formation in the retrosternal area. Therefore, alternative techniques such as partial sternotomy, limited thoracotomy, and subxiphoid and subdiaphragmatic incisions can be used in patients requiring redo cardiac surgery^[3]^. However, it is evident that the use of these techniques requires a longer learning curve and more experience than the standard median sternotomy.

Resternotomy following cardiac surgery is used to treat repeated cardiac diseases and postoperative complications including mediastinitis, sternal dehiscence, and sternal fractures. The choice of sternal opening and closing technique and comorbidities of the patient such as osteoporosis, sex, obesity, COPD, and peri- and postoperative risk factors increase the risk of postoperative complications^[18]^. Of note, sternal repair in the postoperative period dramatically increases the risk of mortality^[19]^.

In our clinical practice, we routinely use CT scan before resternotomy to prevent cardiac and major vascular structures injuries. Before redo cardiac surgery, CT offers certain advantages to the surgeon during surgery, particularly in patients having severe adhesion of the major vascular structures in the retrosternal area and/or right ventricle (i.e., *in situ* right internal thoracic artery used previously for the left coronary system, aortic aneurysms, and/or severely dilated right ventricle). First, a safer operation can be performed in these high-risk patients through peripheral arterial and venous cannulation under cardiopulmonary bypass before resternotomy^[20]^. Second, using a small right anterolateral thoracotomy incision before resternotomy, risky structures for injuries in the retrosternal area (i.e., adhesions in the aorta, patent coronary grafts, and right ventricles) can be removed from the retrosternal area. These strategies may be helpful to prevent catastrophic injuries in the major cardiovascular structures during resternotomy and to reduce mortality^[21]^. However, some authors argue that resternotomy-related injuries have no significant effect on perioperative mortality^[22]^.

In the present study, we used the statistical shape analysis method, which provides additional information by detecting regional and global changes. The main reasons for the widespread use of this method in the field of medicine includes recent advances in imaging technology and the increased interest to investigate the effects of diseases and environmental factors on the structure of the tissues^[23]^. The statistical shape analysis method contributes to the morphometric evaluation of human anatomy^[5]^. In addition, it is a useful tool for postoperative tissue remodeling, which is of clinical relevance in daily practice^[24]^. In previous studies, this method has been successfully utilized in a variety of surgical specialties as a preoperative auxiliary tool^[5,6]^.

Nonetheless, there are some limitations to this study. First, transverse images of only one slice were used to evaluate landmarks at the same level of the chest CT scans (*i.e*., pulmonary artery bifurcation) and only regional deformations were assessed, which led us to examine the relationship between the sternum and the most vulnerable major vascular structures (i.e., ascending aorta and pulmonary artery) located in the anterior and middle-mediastinum of the retrosternal area. However, we were unable to obtain sufficient data regarding the relationship between coronary artery grafts and the retrosternal area, particularly in patients with CABG, due to the lack of slices showing the innominate vein anatomy. In addition, the lack of data regarding whether the pericardium was closed or left open during CABG and the absence of pulmonary artery pressure values might have led to variability in the change of anatomical deformations of the pulmonary artery. There are no available data regarding the mean follow-up for CABG patients. In our study, the patients who underwent CABG operation at least one year ago were included, proving chronic alterations in the retrosternal area during one year. Finally, this study has a single-center and retrospective design with a relatively small sample size. Further large-scale, prospective studies are needed to draw a more definitive conclusion.

On the other hand, the main strenght of the present study is its hypothesis and method in which the statistical shape analysis method was used, since there is a very limited number of studies in the literature evaluating anatomical deformations following cardiac surgery using this method. Hence, it is valuable, as it provides additional information to the body of knowledge on this topic.

## CONCLUSION

In conclusion, our study results show that the retrosternal shape differences and deformations after median sternotomy in CABG patients can be effectively evaluated using a landmark-based geometrical morphometric method based on the topographic distribution of the heart and major vascular tissues. These results also suggest that, among the major vascular structures in the retrosternal area, the main pulmonary artery approximates to the sternum, rather than the ascending aorta, after CABG and, thus, the rate of pulmonary artery injuries may be as high as injuries in the ascending aorta during resternotomy. The present study is valuable as it shows that major cardiovascular structures of the retrosternal area (i.e., innominate vein, superior and inferior venae cavae, right ventricle, left ventricle, CABG grafts, aortic and/or pulmonary conduit grafts) can be evaluated using statistical shape analysis method in patients undergoing CABG and/or other cardiac surgeries with median sternotomy and/or different incisions. We believe it would be a useful guide for further large-scale studies using advanced imaging technology.

**Table:** 

Authors' roles & responsibilities	
MSB	Substantial contributions to the conception or design of the work; or the acquisition, analysis, or interpretation of data for the work; drafting the work or revising it critically for important intellectual content; agreement to be accountable for all aspects of the work in ensuring that questions related to the accuracy or integrity of any part of the work are appropriately investigated and resolved; final approval of the version to be published
GO	Substantial contributions to the conception or design of the work; or the acquisition, analysis, or interpretation of data for the work; drafting the work or revising it critically for important intellectual content; agreement to be accountable for all aspects of the work in ensuring that questions related to the accuracy or integrity of any part of the work are appropriately investigated and resolved; final approval of the version to be published
CK	Substantial contributions to the conception or design of the work; or the acquisition, analysis, or interpretation of data for the work; drafting the work or revising it critically for important intellectual content; agreement to be accountable for all aspects of the work in ensuring that questions related to the accuracy or integrity of any part of the work are appropriately investigated and resolved; final approval of the version to be published
FAB	Substantial contributions to the conception or design of the work; or the acquisition, analysis, or interpretation of data for the work; drafting the work or revising it critically for important intellectual content; agreement to be accountable for all aspects of the work in ensuring that questions related to the accuracy or integrity of any part of the work are appropriately investigated and resolved; final approval of the version to be published
KT	Substantial contributions to the conception or design of the work; or the acquisition, analysis, or interpretation of data for the work; drafting the work or revising it critically for important intellectual content; agreement to be accountable for all aspects of the work in ensuring that questions related to the accuracy or integrity of any part of the work are appropriately investigated and resolved; final approval of the version to be published
EA	Substantial contributions to the conception or design of the work; or the acquisition, analysis, or interpretation of data for the work; drafting the work or revising it critically for important intellectual content; agreement to be accountable for all aspects of the work in ensuring that questions related to the accuracy or integrity of any part of the work are appropriately investigated and resolved; final approval of the version to be published
